# Intramuscular EMG Decomposition Basing on Motor Unit Action Potentials Detection and Superposition Resolution

**DOI:** 10.3389/fneur.2018.00002

**Published:** 2018-01-23

**Authors:** Xiaomei Ren, Chuan Zhang, Xuhong Li, Gang Yang, Thomas Potter, Yingchun Zhang

**Affiliations:** ^1^School of Electrical Engineering and Information, Sichuan University, Chengdu, China; ^2^Department of Biomedical Engineering, University of Houston, Houston, TX, United States; ^3^Guangdong Provincial Work Injury Rehabilitation Hospital, Guangzhou, China; ^4^The Third Xiangya Hospital, Central South University, Changsha, China

**Keywords:** EMG decomposition, segments detection, minimum spanning tree, superposition waveform resolution, pseudo-correlation measure

## Abstract

A novel electromyography (EMG) signal decomposition framework is presented for the thorough and precise analysis of intramuscular EMG signals. This framework first detects all of the active motor unit action potentials (MUAPs) and assigns single MUAP segments to their corresponding motor units. MUAP waveforms that are found to be superimposed are then resolved into their constituent single MUAPs using a peel-off approach and similarly assigned. The method is composed of six stages of analytical procedures: preprocessing, segmentation, alignment and feature extraction, clustering and refinement, supervised classification, and superimposed waveform resolution. The performance of the proposed decomposition framework was evaluated using both synthetic EMG signals and real recordings obtained from healthy and stroke participants. The overall detection rate of MUAPs was 100% for both synthetic and real signals. The average accuracy for synthetic EMG signals was 87.23%. Average assignment accuracies of 88.63 and 94.45% were achieved for the real EMG signals obtained from healthy and stroke participants, respectively. Results demonstrated the ability of the developed framework to decompose intramuscular EMG signals with improved accuracy and efficiency, which we believe will greatly benefit the clinical utility of EMG for the diagnosis and rehabilitation of motor impairments in stroke patients.

## Introduction

Electromyography (EMG) signals carry information regarding the motor unit action potential trains (MUAPTs) generated by the motor units (MUs) that are recruited during muscle contraction. Each MUAPT is made of a series of intermittent discharges that take the form of spatially dispersed individual motor unit action potentials (MUAPs). Intramuscular EMG is commonly acquired by means of indwelling needles or fine wire sensors that provide direct and targeted contact with the musculatures. Clinically, intramuscular EMG is used as a routine method for the electrophysiological examination of neuromuscular symptoms.

EMG decomposition reverses the process of signal generation by separating the de-noised EMG signal into its constituent MUAPTs. This process is accomplished by identifying MUAP waveforms generated by the MUs adjacent to the detection surface and assigning these MUAPs to their corresponding MUAPTs. Characteristic properties of a decomposed MUAP, such as wave shape and firing pattern (1), can provide critical details regarding the health of the nervous system—details that are essential for the clinical diagnosis of neuropathies and myopathies (2–6), and the investigation of the neuromuscular control loop (7). Unfortunately, EMG decomposition is often a difficult and challenging task due to both external interferences, such as poor signal-to-noise ratio (SNR), movement artifacts, shifts in needle position, and so on, and interior challenges, such as waveform variations, intermittent MU firing, and the superposition of multiple MUAPs. EMG decomposition, therefore, requires a complex of advanced signal processing techniques. In the past few decades, many researchers have sought to develop advanced EMG decomposition techniques (8–15). The resultant decomposition methods can be grouped into three categories based on the extent of human interaction: manual, semi-automatic, and automatic (16). Following the manual method, MUAP analysis is performed directly by users who visually inspect and identify the distinctive MUAP patterns (17). This method is time-consuming in practice, highly experience-dependent, and incapable of resolving superimposed waveforms (18, 19). Hence, the development of automatic MUAP extraction methods is imperative to improve the work efficiency and clinical applicability of EMG decomposition. Despite the unremitting effort devoted to the optimization of automatic intramuscular EMG decompositions (5, 16, 20–23), there is still an unmet need for more accurate, complete, and reliable EMG decomposition techniques.

In this paper, we propose a novel intramuscular EMG decomposition framework by advancing the completeness and accuracy of MUAP decomposition. This framework is realized through six stages of analytical procedures: (1) EMG signal de-noising, (2) MUAP segmentation and extraction, (3) MUAPs alignment, feature extraction, and similarity measurement, (4) MUAP clustering and cluster refinement, (5) supervised classification, and (6) superimposed waveform resolution. Following this framework, we have attempted to improve the decomposition performance in four ways. First, we utilized a modified segment extraction scheme that is capable of detecting complete MUAP sets by incorporating amplitude threshold detection and resting segment recognition techniques. Second, single and overlapped MUAP waveforms were identified based on a phasic detection scheme, where phase templates were chosen based on the neurological condition of the tested muscle. The single MUAP segments of each MU underwent a clustering process that markedly reduced the buffer size and processing time required for this task. Third, all recognized single MUAP segments were aligned by centering their main peaks (regardless of polarity). Finally, we resolved superimposed waveforms using a peel-off approach based on measurements of pseudo-correlation (PsC). The performance of the proposed decomposition framework was evaluated using both synthetic EMG signals and real recordings obtained from healthy and stroke participants. Results demonstrated the favorable performance of the developed framework in decomposing intramuscular EMG signals with improved accuracy and efficiency.

## Materials and Methods

### Subjects

Twenty healthy subjects (20–35 years of age, 16 males and 4 females) participated in our data collection. No subject reported any history of neuromuscular diseases. Eight subacute hemiparetic stroke subjects (46–74 years of age, 6 males and 2 females, within 1 month of the ictal event) were recruited from the Third Xiangya Hospital of Central South University in China. The research protocol was approved by the local research ethics committee. All subjects were informed about the purpose and details of the experiment prior to the data collection.

### Data Acquisition

All EMG signals were recorded from the biceps brachii muscle. Subjects were seated in a chair with either the right forearm (for healthy subjects) or the affected forearm (for the stoke patients) supported by a horizontal table. Subjects were then asked to maintain elbow flexion at a 90° angle with their palms facing upward. A conventional needle electrode (9013s0032, Natus Neurology, USA) was inserted into the muscle belly at a depth of approximately 1 cm. Each subject was then asked to perform 10-s constant-force isometric contractions by resisting a load with pre-trained force. Each subject performed three contractions at both mild (3–4 MUs detected) and moderate (6–8 MUs detected) force levels. A 3-min break was provided following each contraction to avoid muscle fatigue. Signal quality and force level were monitored on a real-time display screen with audio feedback. All clinical procedures were performed by an experienced physician (Xuhong Li). The frequency band of the standard EMG instrument was set to 2 Hz–10 kHz. All signals were sampled at 48 kHz and stored for off-line decomposition using an EMG workstation (Dantec Keypoint Focus, Natus Neurology, USA).

### Generation of Synthetic EMG Signals

Synthetic EMG signals are valuable for evaluating decomposition results as, unlike real recordings, the exact firing patterns and waveform templates of the synthetic MUs are known. The use of synthetic EMG signals thereby represents the only way to assess the sensitivity of decomposition algorithms to different parameters. In this study, 5-s segments of EMG signals (sampled at 30 kHz) were generated based on a model proposed by Farina et al. (24), where each segment consisted of one or more channels of synthetic intramuscular EMG recordings. The model was built using a library of real MUAP pools to better approximate biological signals. This library included 40 MUAP waveforms artificially extracted from real EMG signals. Each waveform was expanded by associated Hermite expansion functions in a 16-dimensional space. The firing pattern was generated based on both regular and random firing. The regular firing component of this pattern was created using a mean inter-pulse interval within a stationary-renewal point process, while the embodied random-firing component were determined by uniform random variables (i.e., the positions of the random firing, which were determined by uniform random variables, were used to denote the pattern of the random firing). The synthetic EMG signals were then corrupted by adding random white noise with a variable SNR and background noise. The random noise was simulated as band-pass filtered Gaussian white noise with a zero mean and normally distributed random sequences. The frequency band of the band pass filter was 100 Hz–10 kHz. The background noise was the residual signal obtained by subtracting all recognized active MUAP segments from the original EMG signal. Thirty sets of synthetic EMG signals were generated to evaluate the performance of the proposed decomposition framework.

### De-Noising through Wavelet Filtering and Threshold Estimation

Signal preprocessing followed the methods described in our previous publications (25, 26). Briefly, a wavelet filter was first applied to remove random interference by identifying the wavelets whose frequency range lay outside the 30 Hz–8 kHz window and setting their coefficients to zero. A hard-threshold estimation method was subsequently implemented to eliminate background noise. After performing threshold estimation, the de-noised EMG signals could be reconstructed by an inverse discrete wavelet transform (WT) using modified wavelet coefficients. Additional single-channel-independent component analysis method and digital notch filtration were applied to further remove the residual power-line interference when necessary (25, 26).

### Segmentation and Isolated/Overlapped MUAP Segments Separation

All active MUAP segments were first identified using a modified segmentation scheme. A detection window of 1.25 ms was shifted through the entire EMG signal. A resting segment was recognized if the absolute values of the signal within the window were continuously lower than the pre-set amplitude threshold. Whenever two or more successive resting epochs were detected, the signals spanning between these epochs were extracted as the active segments, in which the signal exceeded this pre-set amplitude threshold. The boundaries of identified active epochs were then spatially expanded by at least 0.2 ms to ensure that the whole MUAP waveform was preserved. This amplitude threshold level was defined as *k* multiplied by the estimated noise power, $\sigma_{n}^{2}$. The noise power of the inactive segments was estimated automatically according to Eqs 1 and 2, based on original EMG signal. The value of *k* was selected by the investigator according to the force level, with a range of 5–8.

(1) $$\sigma_{i}^{2}=\frac{1}{L_{R}}\sum_{k=i}^{i+L_{R}-1}s_{\text{EMG}}^{2}[k],$$

where *L_R_* is the length of a window and the *s*_EMG_[*k*] is the discrete EMG signal. $\sigma_{n}^{2}$ is calculated as the minimum value of $\sigma_{i}^{2}$ according to Eq. 2

(2) $$\sigma_{n}^{2}=\text{min }\sigma_{i}^{2}.$$

Extracted MUAP-containing segments can be either isolated or overlapped. Isolated MUAPs that discharge multiple times can be easily recognized and labeled using clustering methods. Conversely, overlapped MUAP waveforms are created by the partial or full superposition of two or more single MUAPs discharging simultaneously, making the constituent waveforms much more difficult to parse. According to Thornton and Michell (27), MUAPs from a healthy musculature may contain up to four phases while an increase in MUAP phases may be indicative of the MU remodeling period that occurs after pathological denervation. In this study, the subject-specific recognition of isolated/overlapped MUAPs was carried out by assigning a tetraphasic (4-phase) template to the EMG signals from healthy participants and a hexaphasic (6-phase) template to the signals from stroke participants. The phasic properties of the MUAPs are affected by many variables, so it may be improper to assign phasic parameters a fixed value. Thus, the extraction results were evaluated and phasic thresholds were fine-tuned if some of the isolated MUAPs were incorrectly assigned to the overlapped sets. In our experiment, we applied a pentaphasic (5-phase) template for 6 of the 20 healthy data sets and octophasic (8-phase) template for 3 of the 8 stroke data sets. Superimposed waveforms always possess longer durations so, during alignment, segments were zero padded to match the duration of the longest event (22, 28). A very large buffer size would be required if all active segments were to be aligned. To save buffer size and computing time, segment grouping was performed in advance and only the isolated MUAPs were inputted for alignment and clustering. Overlapped MUAPs were not processed until the superimposed waveforms are resolved (see Resolving Superimposed Waveforms Using the Peel-Off Approach Based on Pseudo-Correlation).

Extracted active segments with only one phase or a MUAP duration shorter than 1.5 ms were regarded as invalid and excluded from the detected MUAP set. Finally, the remaining single MUAP segments were retained as a valid set for the following alignment. The beginning points of these active segments, representing the onsets of the MU firing instances, were assembled into a separate one-dimensional array.

### MUAP Waveforms Alignment and Feature Extraction

At this stage, all of the detected MUAP waveforms were aligned with their main peaks (either positive or negative) at the spatial center and shorter waveforms were zero padded so that all segments were of equal length. This alignment scheme can enhance the sensitivity in discerning and grouping MUAPs into their MU origins.

Wavelet-domain features have been shown to improve stability when analyzing EMG signals that are contaminated by high frequency background noise or baseline drift (1, 16). As a result, we implemented WT at the sixth level using the aligned MUAP segment data. The wavelet coefficients from the third through sixth levels of aligned MUAP segments were chosen as the feature space. For WT, we used a compactly supported biorthogonal wavelet base, namely the Daubechies compactly supported wavelet with five vanishing moments, or db5.

After feature extraction, the distance matrix was calculated based on the variance of the error normalized by the sum of the RMS values for the paired segments (20). This is denoted as

(3) $$d(s_{1},s_{2})=\frac{E(e^{2}(n))-E^{2}(e(n))}{\sqrt{E(s_{1}^{2}(n))}+\sqrt{E(s_{2}^{2}(n))}},$$

where *s*_1_(*n*) and *s*_2_(*n*) are the two active segments to be compared and *e*(*n*) is their error signal. The distance measure defined by Eq. 3 was used as a similarity measure for clustering.

### Clustering and Refinement Using the Minimum Spanning Tree (MST) Method

The MUAP set was partitioned into its constituent MUAPTs based on the similarity measure presented above. To do this, we utilized a single-linkage hierarchical clustering algorithm that permits a simple graph-theoretical interpretation, namely the MST method. The MST method, considered best suited for EMG clustering (1), is able to cluster the MUAPs with low variation from one occurrence to the next and does not depend on the presentation order of the samples. We generally set the number of cluster equal to 8–12 depending on the size of the detected single MUAP segments.

A subsequent cluster refining procedure was performed to verify if any potential class should be deleted or subdivided. Clusters with at least three templates were chosen as potential MUAP classes, while those with less than three templates were regarded as invalid MU clusters and excluded. All MUAP segments belonging to these deleted clusters were moved to an unclassified set for subsequent supervised classification. At times, two different MU clusters can be incorrectly assigned to the same cluster due to similarities in their characteristic waveforms. In these cases, the mis-clustered MUs should also be subdivided based on the MST method. MUAPT templates were then calculated as the mean waveforms of each MUAP cluster. After clustering, we obtained the initial sets of MU clusters and the unassigned MUAPs were set aside to be classified in the next step.

### Supervised Classification Based on the Minimum Distance Classifier

At this stage, we used the supervised minimum distance classifier, which is based on measurements of Euclidean distance, to classify the MUAP waveforms in the unassigned candidate set. The classification program was based on the wavelet coefficient features and valid clustering results. During classification, the threshold was set to the lowest mean value obtained from the inter-class distances. Signal instability and electrode movement can cause MUAP shapes to vary from discharge to discharge. Therefore, a weighted averaging technique reported by Zennaro et al. (7) was utilized to adapt the MUAP class template.

### Resolving Superimposed Waveforms Using the Peel-Off Approach Based on Pseudo-Correlation

During muscle contraction, a portion of the entire MU pool is recruited and the intermittent firing pattern of each recruited MU can be extracted as its MUAPT. Multiple MUs that discharge simultaneously or within a very short interval will results in the superposition of MUAP waveforms. Resolving these waveforms is the process of identifying the overlapped MUAP segments and splitting them into their constituent single MUAPs. In our study, a peel-off approach based on PsC was adopted to resolve the superimposed waveforms after the isolated MUAP segments had been successfully classified.

According to Florestal et al. (5), PsC outperforms standard techniques such as cross-correlation-based matched filters and the normalized Euclidean distance. In addition, the PsC between superimposed segments and MU template waveforms can be calculated directly without alignment. Therefore, it is feasible to use PsC as the similarity measure between the superposed segments and MU template waveforms. The PsC between a superimposed segment and a MU template waveform at point *k, PsC_k_*, is defined (5, 29) as

(4) $$PsC_{k}=\frac{\sum_{j=1}^{m}\left(x_{j}y_{k+j}-|x_{j}-y_{k+j}|\text{max}\{|x_{j}|,|y_{k+j}|\}\right)}{\sum_{j=1}^{m}{\left(\text{max}\{|x_{j}|,|y_{k+j}|\}\right)}^{2}},k=1,2,\cdots ,n,$$

where *x_j_* is clustered MU template waveform, *y_j_* the superimposed segment, and *m* and *n* are the size of *x* and *y*, respectively.

The waveform that has the greatest PsC at the point *k* was regarded as the optimal match and was first subtracted from the superimposed segment aligned at point *k*. The matched MUAP waveform and its firing time *k* were then, respectively, assigned to the corresponding MU cluster and associated firing time array. Then the MUAP waveform that had the second greatest PsC was similarly subtracted and assigned. The resolving process continued repeating until segment subtraction resulted in a negative PsC value or an increase in the residual signal energy. In our work, the number of the iterations was experientially set to 3, which was the maximal MUAP number included in the superposed waveform.

### Performance Indices

The following three measures related to the MUAP waveform detection and the EMG decomposition process were used to evaluate the performance of the EMG decomposition system.

#### Detection Ratio

The detection ratio (DR%) was used to measure the rate of the successful detection of active MUAP segments. The DR% is defined as

$$\text{DR}\%=\frac{{\text{NM}}_{\text{detected}}}{{\text{NM}}_{\text{total}}}\times 100\%,$$

where NM_total_ is the total number of MUAP segments—either the size of recruited library of MUs (for synthetic signals) or the number of MUs manually obtained by a neurophysiologist (for real signals)—and NM_detected_ is the number of MUAP segments detected.

#### The Assignment Ratio

The assignment ratio (AR%) measures the rate of MUAP assignment using the proposed EMG decomposition framework. It is defined as

$$\text{AR}\%=1-\frac{{\text{NM}}_{\text{unassigned}}}{{\text{NM}}_{\text{detected}}}\times 100\%,$$

where NM_unassigned_ is the number of MUAP segments not assigned by the EMG decomposition framework and NM_detected_ the total number of MUAP segments detected.

#### The Correct Classification Rate

The correct classification rate (CCR%) assesses the performance of the whole EMG decomposition system. It is defined as the ratio of NM_correct_ (the number of correctly decomposed MUAPs) to NM_detected_ (the total number of MUAPs detected):

$$\text{CCR}\%=\frac{{\text{NM}}_{\text{correct}}}{{\text{NM}}_{\text{detected}}}\times 100\%.$$

## Results

In our study, the DR% of the MUAP detection program for all real and generated EMG recordings reached 100% by using the novel MUAP segment detection scheme.

The complete decomposition of the superimposed waveforms resulted in a marked improvement in the assignment ratio. In this paper, the average assignment ratios (AR%s) of our decomposition system were 99.79% for the synthetic signals, and 98.66 and 91.52% for real recordings from healthy and stroke participants, respectively (listed in Table 1).

**Table 1:** Decomposition results from synthetic and real EMG signals.

EMG data	Synthetic EMG	Real EMG (healthy)	Real EMG (stroke)
DR%	100	100	100
AR%	99.79	98.66	91.52
CCR%	87.23	88.63	94.45

*DR%, detection ratio; AR%, assignment ratio; CCR%, correct classification rate*.

**Figure 1:**
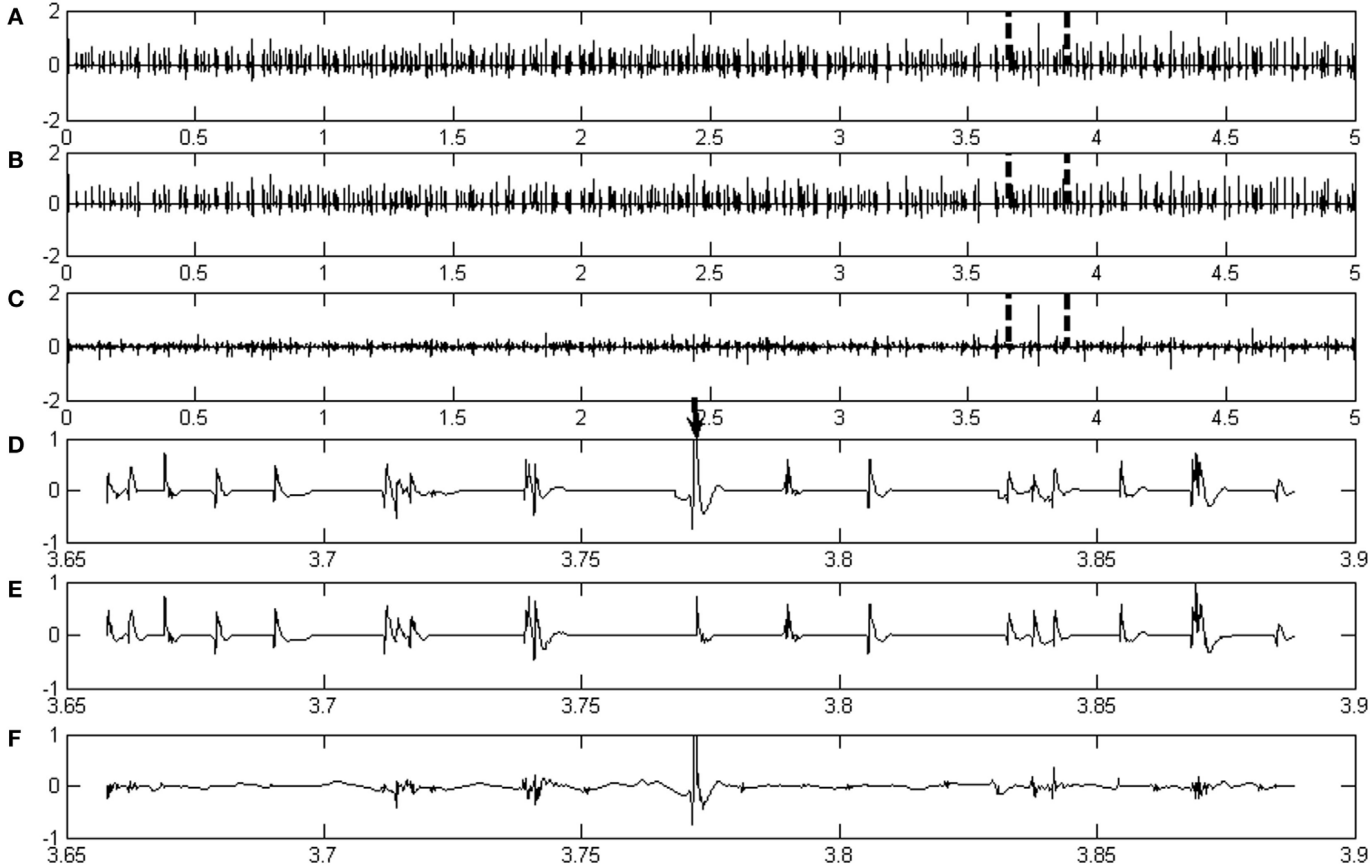
**(A–C)** are, respectively, the real EMG signals from a healthy subject, the assigned motor unit action potential (MUAP) signals, and the residual signal by subtracting the assigned MUAP signals from the original signal. **(D–F)** are, respectively, the details of a section of signals denoted in panels **(A–C)**.

Figures 1A,B show representative examples of real de-noised EMG signals and the correspondingly assigned MUAP signals, respectively. After decomposition, the classified MUAPs were subtracted from the original EMG signal in order to obtain the residual signal, which is shown in Figure 1C. A total of 216 active MUAP segments were detected with an AR% of 99.54% for the signals shown in Figure 1. Only one MUAP segment in this case (marked by the bold arrow in Figure 1D) could not be assigned by the proposed framework.

**Figure 2:**
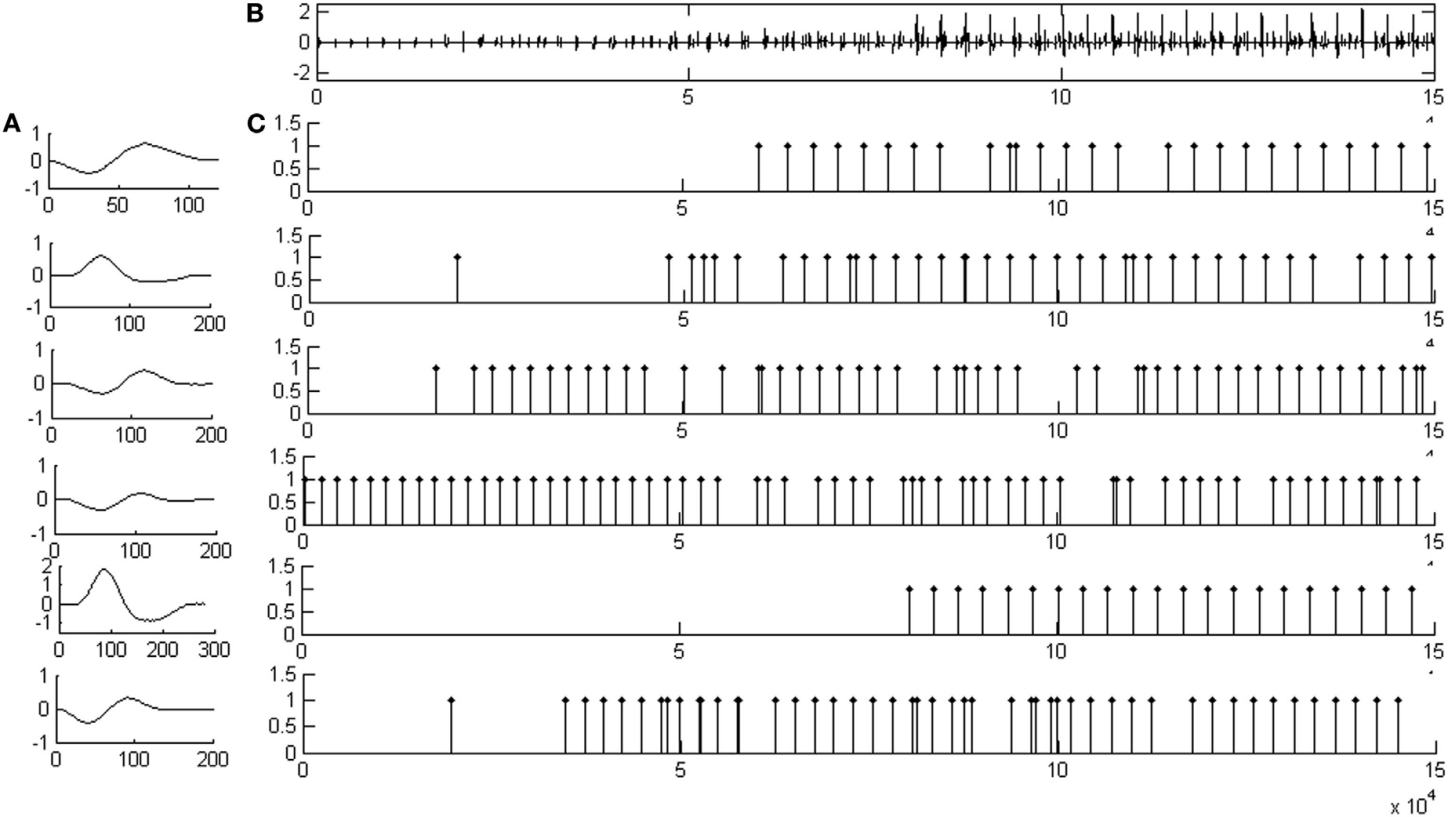
An example of decomposition result based on a synthetic EMG signal. **(A)** The motor unit action potential template waveforms of all motor unit action potential train decomposed from the signal shown in **(B)**. **(B)** The de-noised signal based on a synthetic EMG signal. **(C)** The resulting motor unit (MU) firing patterns for each MU classes identified by the whole decomposition system.

Figure 2 illustrates representative decomposition results based on the synthetic EMG signal, together with the firing patterns and the six identified MUAP template waveforms. Figure 2A shows the MUAP template waveforms decomposed from the signal shown in Figure 2B. Figure 2B depicts the de-noised synthetic EMG signal. Figure 2C demonstrates the corresponding MU firing patterns for each MU class identified by the decomposition framework.

**Figure 3:**
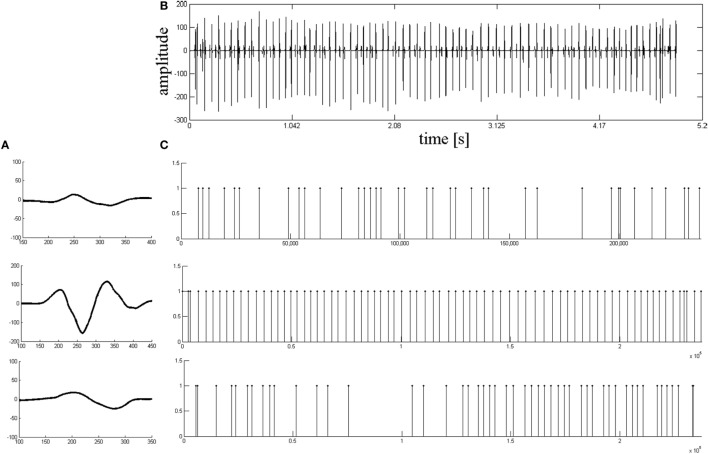
The results of decomposition based on the real EMG signals from a stroke subject. **(A)** Motor unit action potential template waveforms of all motor unit action potential train decomposed from the signal shown in **(B)**. **(B)** The de-noised signal based on the stroke EMG signal. **(C)** The relative motor unit (MU) firing patterns for each MU classes identified by the whole decomposition system.

Figure 3 then provides one example of the decomposition results from a stroke patient. Figure 3A shows the MUAP template waveforms for the three MUAPTs identified from the de-noised signal (shown in Figure 3B). Figure 3C shows the corresponding MU firing patterns for each MU class identified by the decomposition framework.

Table 1 illustrates the decomposition results based on the synthetic and real EMG signals. The decomposition results from the synthetic signals were compared to the known information of the EMG model. The results of the real EMG signals were compared to manual decomposition analysis (assumed gold-standard), performed by an experienced neurophysiologist.

According to Table 1, the CCR% was 87.23% for synthetic EMG signals, and 88.63 and 94.45% for real recordings from healthy subjects and stroke patients, respectively.

**Figure 4:**
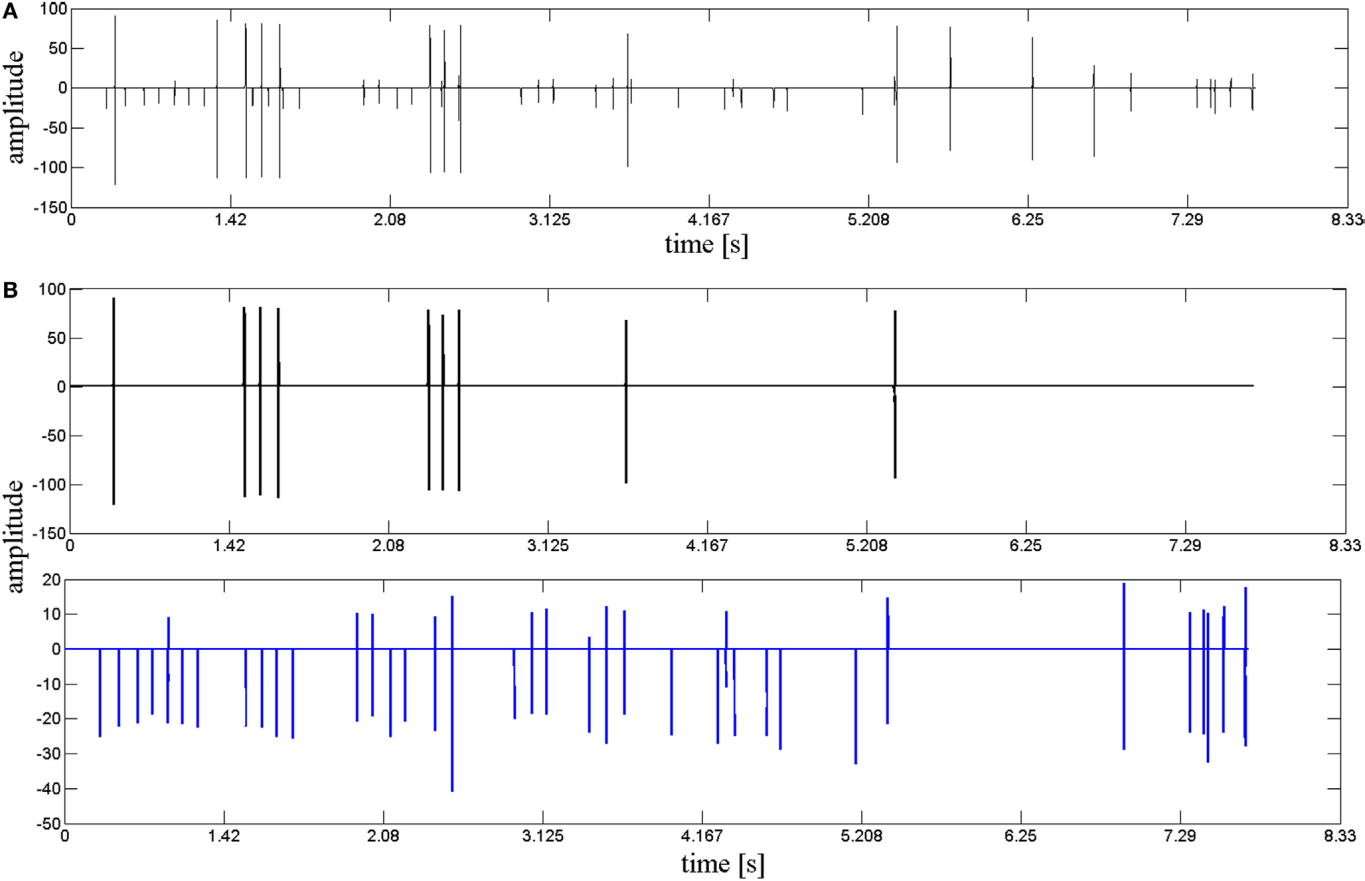
The result of the cluster refinement. **(A)** A motor unit (MU) firing incorrectly clustered after original clustering. **(B)** Two correct MU firings (displayed, respectively, in black and blue) subdivided by cluster refinement.

Accurate clustering results are critical to the decomposition performance. In our study, we performed a cluster refining step to improve the accuracy of results. Cluster refinement in this case included deleting invalid clusters and subdividing one incorrectly identified cluster into two or more clusters. Figure 4A demonstrates a MU cluster after preliminary clustering, where two clusters were found incorrectly grouped due to similarities in their waveforms. Further clustering refinement was performed based on methods described in Section “Clustering and Refinement Using the Minimum Spanning Tree (MST) Method,” where an MST method was adopted to further subdivide the erroneous cluster into two separate MU clusters, shown as in Figure 4B. Following this method, the erroneous cluster was successfully subdivided into two separate MU clusters.

The whole analysis process was conducted using a custom MATLAB script and performed on 2.5 GHz Intel i7 desktop computer. The average processing time for decomposing a 10-s-long EMG data was approximately 15–20 min.

## Discussion

EMG decomposition has been widely employed to provide information of alterations in motor unit characteristics in stroke patients (30). Achieving the complete and accurate motor unit firing pattern is vital for the understanding of pathological alterations in patients, as well as for clinical diagnosis and management. Therefore, the goal of this EMG decomposition framework is to identify complete MUAP segments in the EMG signal and classify them accurately into their constituent MUAPTs. Both the template waveforms and firing rates of the MUAPTs are largely dependent on the configuration of the needle electrode, the relative position of electrode to the muscle fibers, the level of contraction, and the pathological condition of the muscle. In this study, we developed an EMG signal decomposition framework based on a novel MUAP segmentation method and the resolution of superimposed MUAP waveforms. Results showed strong decomposition performance with high values for DR%, AR%, and CCR%. It should be noted that the CCR%s obtained for the stroke subject EMG signals were higher than those found in the other two conditions (signals from simulated and healthy subjects), signifying a potential clinical application for this method in the assessment of neurogenic disorders. This is probably due to a well-studied denervation process that occurs in post-stroke patients (30). Compromised MU recruitment in these cases often leads to sparser MU firing patterns and consequently higher identification accuracy.

A high DR% value is critical, as the successful extraction of MUAPs greatly impacts subsequent decomposition procedures and, consequently, the AR% and CCR%. All active MUAP segments and resting segments comprise the whole EMG signal. Due to signal disparities, direct identification of MUAP segments is more difficult than the identification of the resting epochs. Thus, we utilized a wavelet hard-threshold estimation technique to attenuate instrumental and background noises, then applied a novel segmentation scheme based on resting segment detection. All active MUAP segments were further detached by subtracting rest segments from the original signal. The employment of a modified segmentation scheme greatly enhanced the performance, achieving a DR% value of 100% and demonstrating the complete detection of all active MUAP segments. Conventional segmentation methods often fail to identify some active MUAPs, even when using different rigid detection thresholds, because of the abnormal waveform complexity (26). Therefore, this complete detection of MUAPs—reaching a DR% of 100%—is unlikely to be achieved using conventional approaches.

Motor unit action potential segments detected in the aforementioned manner can be either isolated MUAP segments or superimposed MUAP waveforms. Then isolated/overlapped MUAP segments were separated based on either tetra- or hexaphasic waveform recognition methods. Segments with more than four MUAP phases for healthy subjects or six phases for stroke subjects were generally recognized to contain superposed MUAPs and separated for further analysis to resolve the superposition. Thus, by grouping segments based on their isolated MUAP characteristics in the first instance, the efficiency of the decomposition system was improved greatly. The implemented main peak alignment method further served to improve the methodical distinguishability of MUAPs originating from different MU clusters, resulting in more accurate and efficient results.

In our study, the MUAP waveforms were clustered using a single-linkage hierarchical clustering algorithm. This technique is suitable for the clustering of MUAPs with slow variation and does not depend on the presentation order of the samples. However, the results of clustering are very sensitive to the value of the discriminatory threshold. Clustering results were, therefore, verified through visual inspection. Invalid clusters were excluded or subdivided again using the MST algorithm to ensure that all final clusters are valid. In addition, the fuzzy *k*-means algorithm is based on the minimization of a global cost function, which is related to its classification ability. It is our ongoing effort to further integrate these two clustering algorithms and, in doing so, achieve optimal clustering results.

To obtain a thorough EMG decomposition, superimposed waveforms need to be resolved into their constituent MUAPs. This stage is the most time-consuming and critical procedure in the whole EMG decomposition framework. As it is essential to obtain a high-level AR% and complete information, the consistent achievement of AR%s over 90% by the new framework represents a marked improvement over the 67% classification rate achieved by our previous methods (25). Two typical types of superposed waveform resolution approaches have been commonly employed: peel-off and modeling (31). In this study, we resolved superimposed waveforms using the peel-off approach based on a pseudo-correlation method that improves resolution efficiency. Despite its apparent efficacy, it should be noted that the peel-off method is incapable of identifying MUAP waveforms that superimpose in a destructive manner (1). Waveform resolution based on modeling can yield a more accurate separation but is also more time-consuming. Therefore, identifying a method that improves the efficiency and accuracy of superimposed waveform decomposition remains a focus for further exploration.

In summary, an effective EMG decomposition framework was developed. First, we utilized a novel MUAP segment extraction method to detect all active MUAP segments. This procedure was based on amplitude threshold detection and resting segment recognition. We then grouped the MUAP segments into single and overlapped waveforms using tetraphasic or hexaphasic detection schemes to save buffer size and improve computational efficiency. Third, all recognized single MUAP segments were aligned with the main peak at the center for the effective assessment of waveform similarities. Finally, we resolved superimposed waveforms using a peel-off approach based on a measure of PsC. By incorporating multiple analytical approaches, this developed EMG decomposition framework achieves accurate and complete results without hampering computational speed, which we believe will greatly benefit clinical EMG utilities.

## Ethics Statement

The study protocol was approved by the West China ethics committee of Sichuan University and the Institutional Review Board of Third Xiangya Hospital, Central South University.

## Author Contributions

XR led the development of the proposed decomposition algorithm, participated in healthy subject experiment, data acquisition and analysis, and paper writing. CZ participated in stroke patient experiment, data acquisition, and paper writing; XL participated in stroke patient recruitment, data acquisition, and analysis; and GY participated in algorithm development, data acquisition, and data analysis. TP participated in data analysis and the article polish. YZ participated in study design, algorithm development, data acquisition and analysis, and paper writing.

## Conflict of Interest Statement

The authors declare that the research was conducted in the absence of any commercial or financial relationships that could be construed as a potential conflict of interest.
